# Comment on Senthilkumaran et al. Bilateral Simultaneous Optic Neuritis Following Envenomations by Indian Cobra and Common Krait. *Toxins* 2022, *14*, 805

**DOI:** 10.3390/toxins16030112

**Published:** 2024-02-22

**Authors:** Maarten B. Jalink

**Affiliations:** 1 Department of Ophthalmology, Central Military Hospital, Lundlaan 1, 3584 EZ Utrecht, The Netherlands; maartenjalink@hotmail.com; 2 Department of Ophthalmology, University Medical Center Utrecht, Heidelberglaan 100, 3584 CX Utrecht, The Netherlands

**Keywords:** snakebite, optic neuropathy, antivenom

It is with interest that I read the case report by Senthilkumaran et al. [1], in which the authors described two cases of severe optic neuropathy following envenomation by an Indian cobra (*Naja naja*) and a common krait (*Bungarus caeruleus*). Both patients were treated with antivenom due to systemic envenomation, but developed bilateral optic neuropathy four and five days after the bite regardless. There was severe visual loss and papilledema with hemorrhages; the diagnosis of optic neuropathy (or optic neuritis) was established using MRI. Both patients regained full visual acuity after a high dose of intravenous methylprednisolone. The authors present various possible mechanisms for the cause, including “allergic reactions to antivenoms” amongst other causes. I would like to take the opportunity to present a few arguments from other studies that suggest antivenom as a cause for the reported symptoms.

In our review of the online available literature [2], ten other case reports of optic neuropathies after a snakebite can be found [3,4,5,6,7,8,9,10,11,12]. Nine describe a similar syndrome of severe bilateral vision loss and papilledema with hemorrhages, several days (mostly six) after initial envenomation. Symptoms of neurotoxicity after snakebite envenomation usually develop more quickly (several minutes up to nineteen hours [13]). A conflicting temporal association between the bite and the onset of ocular symptoms would make a direct effect of the venom less likely. Various snakes are described in the literature; three patients were envenomated by elapids [7,8,11] and four by vipers [3,4,9,10]. While elapid venom is mostly neurotoxic, viper venom is predominantly hemotoxic. Due to species-specific differences in venoms, venom as a common cause for optic neuropathy is therefore questionable. Antivenom, however, is a common cause; the reported patients received intravenous antivenom as part of the initial treatment, before the development of ocular symptoms [3,4,5,6,7,8,9,10,11]. Interestingly, in one of the reported cases, the offending snake was discovered to be non-venomous after the antivenom had already been administered [5]. This patient, too, developed visual deterioration with papilledema six days after antivenom was given. The same syndrome has even been described in a case of scorpion envenomation, in which a (similar) antivenom was given to a patient without signs of systemic involvement [14]. She developed bilateral optic neuropathy after five days as well.

Though there are a few cases of optic neuropathy after a snakebite, antivenom could be one of the probable causes. However, it is hard to draw definitive conclusions, and other causes should not be overlooked. Inflammation caused by a snakebite, triggering an aberrant immune response, could be another cause explaining the above cases. Since antivenom is given in a majority of venomous snakebites, this could be a probable cause of bias in this regard. Also, in most reports, there is no clear description of the used antivenom. Authors are encouraged to specify the type (e.g., serum immunoglobulins or immunoglobulin fragments), dose, and producer of antivenom in future reports. This information can contribute to the development of a new generation of antivenoms.
